# Dietary patterns of a versatile large carnivore, the puma (*Puma concolor*)

**DOI:** 10.1002/ece3.9002

**Published:** 2022-06-29

**Authors:** Harshad Karandikar, Mitchell W. Serota, Wilson C. Sherman, Jennifer R. Green, Guadalupe Verta, Claire Kremen, Arthur D. Middleton

**Affiliations:** ^1^ Department of Environmental Science, Policy & Management Mulford Hall University of California Berkeley California USA; ^2^ Institute for Resources, Environment and Sustainability University of British Columbia Vancouver British Columbia Canada; ^3^ Department of Zoology Biodiversity Research Centre University of British Columbia Vancouver British Columbia Canada

**Keywords:** diet, feeding ecology, large carnivore, predation, puma

## Abstract

1. Large carnivores play critical roles in terrestrial ecosystems but have suffered dramatic range contractions over the past two centuries. Developing an accurate understanding of large carnivore diets is an important first step towards an improved understanding of their ecological roles and addressing the conservation challenges faced by these species.
2. The puma is one of seven large felid species in the world and the only one native to the non‐tropical regions of the New World. We conducted a meta‐analysis of puma diets across the species’ range in the Americas and assessed the impact of varying environmental conditions, niche roles, and human activity on puma diets. Pumas displayed remarkable dietary flexibility, consuming at least 232 different prey species, including one Critically Endangered and five Endangered species.
3. Our meta‐analysis found clear patterns in puma diets with changing habitat and environmental conditions. Pumas consumed more larger‐bodied prey species with increasing distance from the equator, but consumption of medium‐sized species showed the opposite trend.
4. Puma diets varied with their realized niche; however, contrary to our expectations, puma consumption of large species did not change with their trophic position, and pumas consumed more small prey and birds as apex predators. Consumption of domestic species was negatively correlated with consumption of medium‐sized wild species, a finding which underscores the importance of maintaining intact native prey assemblages.
5. The tremendous dietary flexibility displayed by pumas represents both an opportunity and a challenge for understanding the puma’s role in ecosystems and for the species’ management and conservation. Future studies should explore the linkages between availability and selection of primary and other wild prey, and consequent impacts on predation of domestic species, in order to guide conservation actions and reduce conflict between pumas and people.

## Graphical abstract

We conducted a meta‐analysis of puma diets across the species’ range in the Americas and assessed the impact of varying environmental conditions, niche roles, and human activity on puma diets. Our meta‐analysis found clear patterns in puma diets with changing habitat and environmental conditions. Puma diets also varied with their realized niche. The tremendous dietary flexibility displayed by pumas represents both an opportunity and a challenge for understanding the puma's role in ecosystems and for the species’ management and conservation.

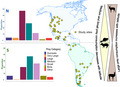

## 1 INTRODUCTION

Large carnivores play critical roles in shaping and regulating ecosystems. In addition to the well‐documented trophic cascade impacts on food webs in ecosystems, recent research has highlighted the impacts of these species on a wide range of ecological processes, including limiting the spread of disease, carbon sequestration, and regulating biogeochemical cycles (Estes et al., 2011; Pauli et al., 2018). Despite this, large carnivores are among the most threatened taxonomic groups across the world (Ripple et al., 2014). Most large carnivore species across the world have seen significant population declines and range contractions, with intact carnivore guilds limited to only about a third of the world's land area (Wolf & Ripple, 2017). Although most of these species are now legally protected and are the focus of conservation actions across the globe, the severity and widespread nature of the threats faced by these species continues to threaten the persistence of many large wild carnivore populations (Ripple et al., 2014). Understanding carnivore ecology and behavior is critical to ensure the success of conservation efforts for these species and maintaining functional large carnivore populations that regulate critical ecological interactions.

Diets are an important component of a species’ ecology and function and offer vital information on important biological parameters including niche breadth, trophic specialization, and prey selection (Monterroso et al., 2019). Diets and dietary flexibility, especially in the case of species such as obligate carnivores that depend on specific food categories, may also be the limiting factor in the ability of a species to adapt to changing environmental conditions. Prey depletion is one of the biggest threats to large carnivore populations across the world (Wolf & Ripple, 2016), and the reduced availability of large‐sized prey may have played a key role in some of the late Quaternary extinctions (Meachen‐Samuels & Van Valkenburg, 2010). Plasticity in large carnivore diets and predation behavior is, however, rarely systematically assessed and understood, especially in the case of ambush predators that attack large prey in complex terrain (Sunquist & Sunquist, 2019; Williams et al., 2014). Understanding diets and dietary flexibility may also help in understanding indirect and cryptic interactions, as well as highlighting the risk of secondary extinctions (Brodie et al., 2014). From an ecological perspective, greater flexibility in a species is likely to increase the context dependency of its ecological impacts, for example, on lower trophic levels. From a conservation point of view, greater flexibility is likely to increase resilience to human disturbance, given that more flexible species are likely to survive in increasingly fragmented landscapes (Devictor et al., 2008). Although many large felids display high levels of resilience by adapting to and even thriving in many highly human‐modified environments, including high‐intensity agriculture (Warrier et al., 2020) and dense urban areas (Athreya et al., 2013; Benson et al., 2020), this flexibility also brings them into close contact with humans, resulting in increased negative interactions with people (Athreya et al., 2016). This is especially important as carnivores around the world recolonize large parts of their former range after serious declines and population extirpations in the nineteenth and twentieth centuries (Chapron et al., 2014; Gompper et al., 2015; Miller et al., 2013), due to a combination of factors, including changing attitudes, enhanced legal protections, and improved practices that facilitate coexistence. This range expansion and recolonization can thus result in unintended or unexpected consequences (Pauli et al., 2018). A systematic understanding of large carnivore diets can thus offer important insights into ecosystem functioning and inform policy, conservation, and wildlife management actions.

The puma (*Puma concolor*) is one of seven large felid species in the world and the only one native to the non‐tropical regions of the New World. Pumas are found in a diverse range of habitats and environments, from mountainous temperate regions to tropical areas, and from wilderness to areas with high levels of human use (Benson et al., 2020). Pumas are apex predators in large parts of their natural range, especially in southern South America, but are subordinate to other large predators including gray wolves (*Canis lupus*), grizzly bears (*Ursus arctos*), and jaguars (*Panthera onca*) in North and Central America and the tropical regions of South America (Elbroch & Kusler, 2018). The species plays an important ecological role with critical trophic cascade impacts documented or hypothesized in several settings (Leempoel et al., 2019; Ripple & Beschta, 2006; Wang et al., 2015), including on other key functional groups such as scavengers like the Andean condor (*Vultur gryphus*) (Perrig et al., 2017), and a multitude of other important biotic relationships (LaBarge et al., 2022). Despite being considered as the archetypical ambush hunter, some studies suggest a certain degree of flexibility in puma hunting styles, habitat needs, and diets (Anderson, 1983; Hornocker & Negri, 2010; Iriarte et al., 1990), which may have been instrumental in the species’ recolonization of several parts of its former range despite widespread persecution resulting in precipitous declines in puma numbers and range contractions in the twentieth century (Mazzolli, 2012; Walker & Novaro, 2010). Despite these range recoveries, puma populations continue to see an overall declining trend (Nielsen et al., 2015). A systematic analysis of the dietary patterns and the dietary flexibility displayed by the species can offer additional insights that can inform conservation actions for a species that is considered as a high conservation priority felid despite its wide‐ranging status (Dickman et al., 2015).

We conducted a systematic review of literature on puma diets across the species’ geographical range in the Americas, with the primary objective of understanding puma dietary plasticity and its possible ecological and conservation implications. Specifically, we analyzed puma dietary patterns across changing habitats, environmental conditions, trophic dynamics, and human use of the landscape and tested the following hypotheses and predictions:

1. Puma diets will change across latitudes, biomes, and continents due to differences in prey diversity and availability, primarily greater availability of larger prey with increasing latitude (Blackburn & Hawkins, 2004), and regional differences in prey availability.
  - Consumption of larger prey species will increase with increasing latitudes (as reported in Iriarte et al., 1990), whereas consumption of medium and smaller‐sized prey will be greater in tropical biomes (Blackburn & Hawkins, 2004)
  - Consumption of larger species will be greater in North America, whereas the consumption of small mammals and rodents will be greater in South America (Iriarte et al., 1990)
  - Dietary diversity will be greater in the tropical biomes and in South America due to the greater availability and diversity of small prey species (Iriarte et al., 1990; Murphy & Ruth, 2010)
2. Puma diets will change depending on the species’ trophic position as an apex or subordinate predator
  - The consumption of larger prey species will be greater in ecosystems where the puma is an apex predator (as hypothesized in Iriarte et al., 1990) due to the absence of competition with larger predators
  - Dietary diversity will be greater in ecosystems where the puma is a subordinate predator due to competition with larger predators for larger prey
3. Puma diets will change due to the influence of anthropogenic disturbances (as hypothesized for gray wolves in Newsome et al., 2016).
  - In areas with increased human footprint on the landscape, pumas will consume more domestic species and smaller‐sized prey
  - The consumption of domestic species will be greater when the consumption of large wild prey is lower
  - Puma diets will change over time to include greater proportions of domestic species due to changes in human impact on landscapes

## 2 METHODS

### 2.1 Literature review and data collection

We searched the databases Web of Science, Scopus, and Google Scholar for studies on puma diets and food habits, using two search strings: (1) puma OR "mountain lion" OR cougar AND diet, and (2) puma OR "mountain lion" OR cougar AND food. For the first 500 results for each search, we reviewed the study abstract to determine whether the study was focused on or included data on puma diets. A total of 68 studies were identified through this search process. An additional 35 studies were found by reviewing studies cited in the shortlisted studies and by reviewing book chapters and personal bibliographies of the authors. While the primary literature search was conducted between March and April 2020, we also included subsequently published articles that were relevant to the analysis. Studies from this list were subsequently added to the primary dataset if they met all of the following criteria: (1) diet data were stated as frequency of occurrence (FO), defined as the proportion of scats or stomachs that included a particular prey species or prey category, or as percentage of occurrence (PO), defined as the proportion of kills, scats or stomachs that comprised of a particular prey species or prey category; or where FO or PO values could be easily calculated from the data, (2) sample size was at least 5 units (scats, stomachs, or clusters), (3) samples were collected in more than one season, (4) values for most dietary categories (explained below) were clearly stated or could be calculated, and (5) the study unambiguously distinguished puma‐specific samples from those of any sympatric felids. After filtering for these criteria, 73 studies were initially retained for our analysis (Appendix S1). If a study reported separate data from multiple locations or time periods, each instance was considered an independent data point unless these locations or time periods were spatially or temporally adjacent. If a study reported data from multiple locations without a clear physical gap between these locations (for example, separately reported data from a protected area and the adjoining working landscape) or from consecutive calendar years or seasons, these data were combined and considered as a single data point.

For each retained study, we determined the location of the study area, study length in months, median study year, study area biome, continent (North, Central or South America, with Central America defined as the region south of Mexico to the southern boundary of Panama), the kind of samples used for diet assessment (scats, stomachs, or clusters), sample size, diet metric(s) used (FO and/or PO), a value for the human footprint index (explained below) (Venter et al., 2016, 2018), a value for dietary diversity and whether pumas were apex predators or sympatric with other large predators in the study area (Elbroch & Kusler, 2018). Studies from Central America were subsequently combined with studies from North America for analyses. If the coordinates of the study location were not stated, we used Google maps to visually estimate the centroid for the study area based on the description in the study. The study area biomes were determined using the classification in Olson et al., 2001, and subsequently pared down to five major biome categories: grasslands, tropical forests, temperate forests, Mediterranean habitats, and deserts. Human impact values were determined by using the human footprint index (Venter et al., 2016, 2018). The human footprint index value was determined by calculating the average value in a 50 km buffer around the study area centroid using the *raster* package (Hijmans & van Etten, 2016) in R (Newsome et al., 2016), using the values from Venter et al. (2018), which offers a high‐resolution index of human impact and influence, calculated using a variety of indicators such as population densities, linear infrastructure, and land use types. Dietary diversity was estimated using the standardized Levin's measure of niche breadth (Hurlbert, 1978; Levins, 2020).

We classified puma dietary data by assigning each consumed prey species to one of seven food groups, adapted from Newsome et al. (2016): (1) domestic species, irrespective of body mass or taxonomic class, (2) very large wild prey (>130 kg), (3) large wild prey (23–130 kg), (4) medium wild prey (3–22 kg), (5) small wild prey, including small rodents (0.1–2 kg), (6) birds, irrespective of body mass, and (7) other or unidentified species. We used average body mass values from the Ecological Society of America (ESA) Pantheria database (Jones et al., 2009) and rounded values to the closest integer. We calculated group values by summing values for all species for the group. If a study included only FO data, group PO values were calculated from the group FO values by calculating the group FO to total FO ratio (Tirelli et al., 2019; Wang, 2002). If a study stated separate dietary values for each season or for male, female, and subadult pumas, we used weighted averages to determine overall values.

### 2.2 Data checks

FO is the most commonly used metric for measuring carnivore diets (Klare et al., 2011). A large proportion of the available large carnivore dietary studies are, however, based on identified carnivore kills that can only offer PO data. Although many dietary reviews exclude studies without FO data (Doherty et al., 2015, 2019), this approach eliminates many studies and significantly reduces sample sizes. Previous research contends that both kill‐based and scat‐based methods offer similar results in terms of prey composition (Perilli et al., 2016; but see Ackerman et al., 1984 and Klare et al., 2011). In addition, studies based on GPS cluster investigations have reported significant numbers and proportions of small prey in large carnivore diets (Allen et al., 2015; Pitman et al., 2014). To check for the effect of the metric used (FO or PO) on puma diet composition and in order to avoid bias due to small sample sizes, we used the approach of Newsome et al. (2016). First, using the mean diet category values for each biome, we used a Mantel test (Mantel, 1967) with 1000 iterations to compare the full dataset with a subset of the full dataset that only included studies that used FO as a metric. Next, as an additional check, we used a multivariate analysis with 1000 iterations with the *mvabund* package (Wang et al., 2012) to test for the effect of diet metric and sample size on the eight food groups. Sample size values included the number of analyzed scats, stomachs, colons, and investigated kills, including those found at GPS clusters, as reported in the study.

The Mantel test revealed that the two datasets were strongly and significantly correlated (*r* = .9, *p *< .001), suggesting that the FO subset mirrors the full dataset. The multivariate analysis of the full dataset, with the diet categories as the response variables, revealed a significant effect of both the diet metric and sample size; therefore, we tested whether the diet metric was still significant after omitting studies with a sample size less than 20. The analysis with a minimum sample size of 20 showed no significant effect of the diet metric at the multivariate or univariate level. This dataset was thus used for all subsequent analyses, consisting of 71 independent data points from 62 unique studies.

### 2.3 Analyses

We used separate multivariate (i.e., multiple response variables) models with a negative binomial distribution to assess the role of differences in latitudes, biomes, continents, niche roles, human impacts on the landscape, and time on puma diets. To account for the effects of multiple testing, we report adjusted p‐values for univariate tests and use a cutoff value of 0.05 to report significant results. Diagnostic plots and checks outlined in Wang et al. (2012) were used to confirm compliance with model assumptions. In each model, the PO values of the seven food categories were the response variables and the factors that were hypothesized to impact these variables, including latitudes, biomes, continents, niche roles, and human impacts, were the predictor variables. For example, in the model assessing the relationship between latitudes and puma diets, the seven food category values were the response variables, whereas latitude was the predictor variable. Absolute latitude values were used to understand how diets changed with increasing distance to the equator. To assess the effect of time, the median study year was used. All variables were scaled and centered. All multivariate analyses were done using the *mvabund* R package (Wang et al., 2012). We used one‐way anova to assess the relationship between dietary diversity and biomes and a Mann‐Whitney *U* test to understand the relationship between dietary diversity and continents and between dietary diversity and niche role.

## 3 RESULTS

**FIGURE 1:**
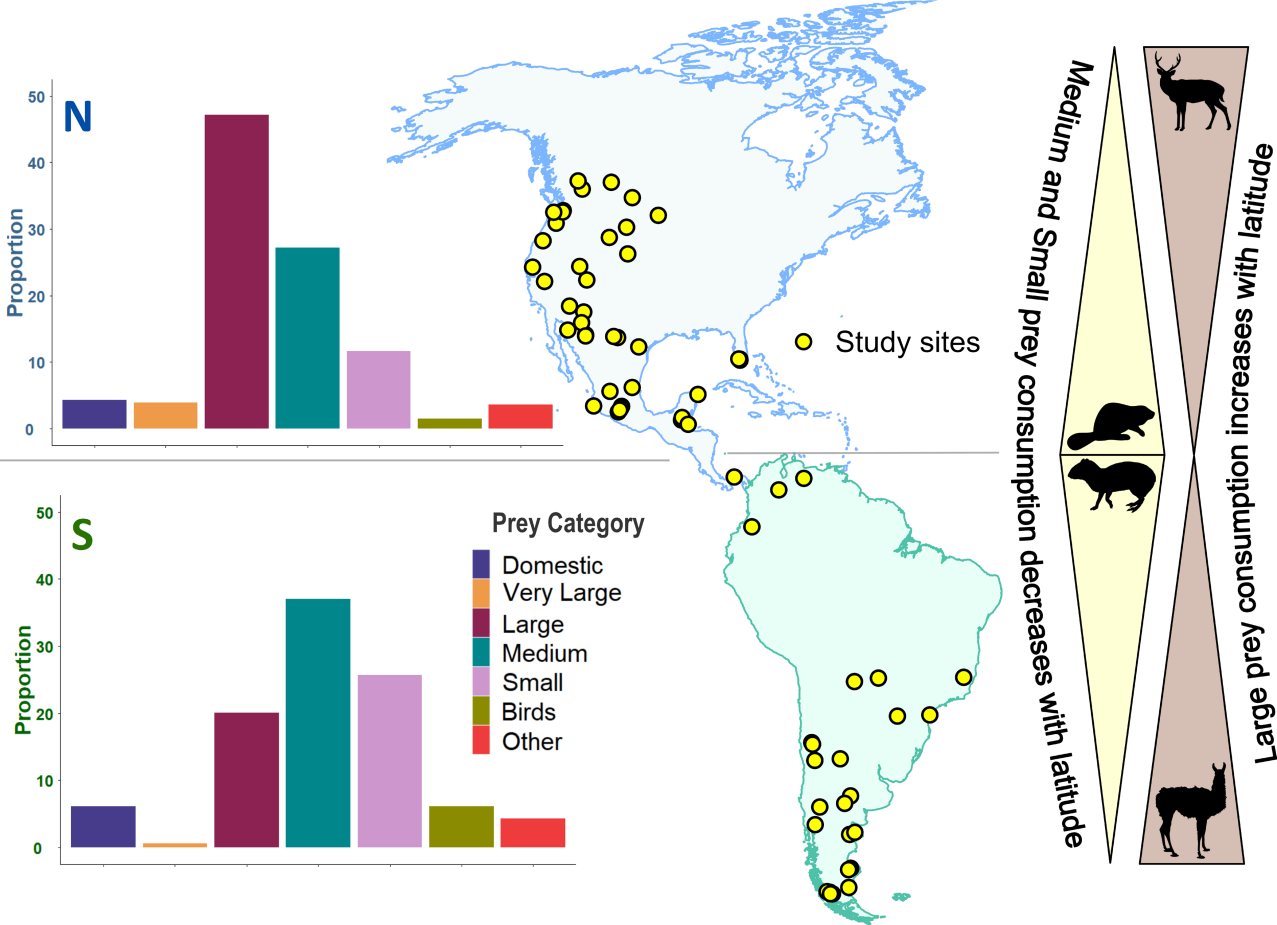
Puma diet composition in North (upper panel) and South America (lower panel). Pumas consumed significantly more large prey in North America compared with South America. Puma diets comprised more large prey at higher latitudes and more medium‐sized prey at lower latitudes

Pumas preyed upon at least 232 unique species, including 19 very large and large wild mammals, 67 medium‐sized wild mammals, 92 small wild mammals, 30 birds, and 8 domestic species, including 1 Critically Endangered and 5 Endangered species (Appendix S2). Out of the 99 independent data points from the 73 studies in the initial dataset, 50 were from North America, 44 from South America, and 5 from Central America. An important point to note here is the absence of studies from a large part of the puma range in the Amazon (Figure 1). Of these 99 independent data points, 68 consisted of diets assessed from an analysis of scats, 22 from cluster studies, 7 from stomach contents, 1 from colon contents, and 1 study that used data from scats and stomachs. The number of species across North and South America was evenly distributed, with 125 species consumed by pumas in North America compared to 117 in South America. The mean sample size for the dataset used for the analyses (excluding studies with sample sizes smaller than 20) was 186.87 ± 25.44 samples (mean ± SE). The mean study duration was 49.99 ± 5.06 months, excluding one study that did not report a study length.

### 3.1 Differences across latitudes, biomes, and continents

**FIGURE 2:**
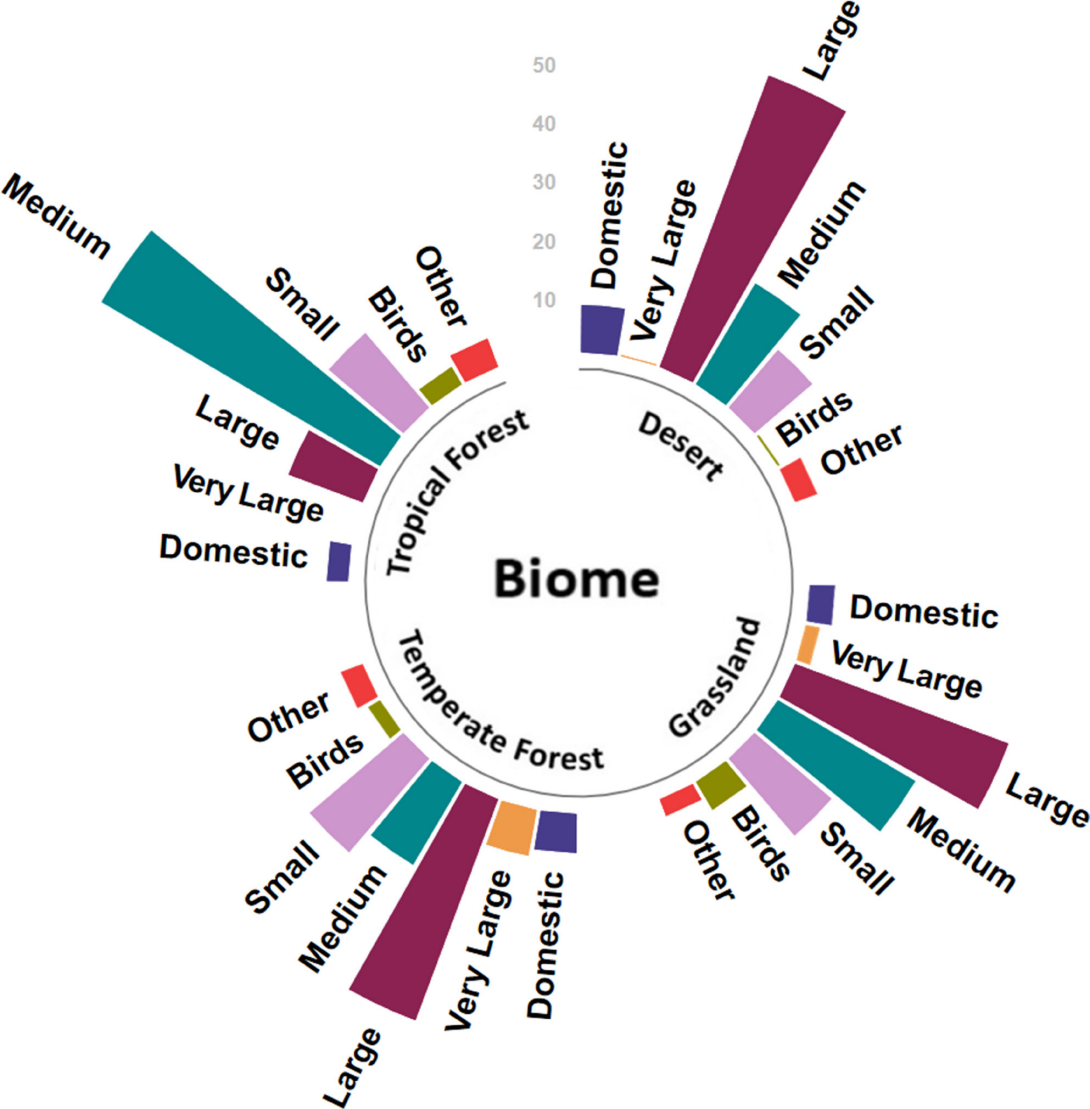
Consumption of prey across prey categories in the major biome groups. Except tropical forest biomes, where medium‐sized prey dominated puma diets, large species were the dominant prey category

We found evidence to support our hypothesis that puma diets change with latitudes, biomes, and continents. Puma diets changed significantly with changes in latitude (F_1,69_ = 42.51, *p* < .001). Univariate tests showed a significant effect of latitude on consumption of very large (F = 15.76, *p* < .001), large (F = 8.87, *p* = .006), and medium (F = 15.26, *p* < .001) mammals, but not on domestic species, small mammals, and birds. In line with our first prediction, consumption of very large and large species increased with increasing latitude, while that of medium‐sized species showed a decreasing trend with increasing latitude (Figure 1). Puma diets also varied significantly across biomes (F_4,66_ = 93.82, *p* < .001), and univariate tests indicated significant effects of biomes on very large (F = 17.25, *p* = .008), large (F = 21.89, *p* = .001), and medium (F = 27.08, *p* < .001) mammals and birds (F = 13.15, *p* = .036). Medium‐sized species were consumed the most in tropical forests and the least in temperate forest biomes (Figure 2). Significant differences were found between puma diets in North and South America (F_1,69_ = 39.91, *p* < .001), with univariate differences found for large (F = 12.87, *p* = .002) and small mammals (F = 8.69, *p* = .009) and birds (F = 12.52, *p* = .002), but not for any of the other groups. Large mammal consumption was higher in North America, while small mammal consumption was higher in the south, confirming our second prediction. Puma diets in North America were biased toward large wild prey, with medium‐sized wild prey the next most consumed category, as compared to South America, where medium‐sized prey constituted the largest food group, with large‐medium wild prey and small mammals constituting other important groups (Figure 1). Dietary diversity did not change across biomes (F_10,60_ = 1.07, *p* = .402) or between North and South America (*p* = .073).

### 3.2 Impact of niche roles on puma diet

We found partial support for our hypothesis that the niche role played by pumas in the ecosystem impacted puma diets, but did not find support for our prediction that consumption of larger prey species will be greater in ecosystems where the puma was an apex predator. Puma diets changed significantly in regions where the species was an apex predator, compared with regions where they were subordinate to other large predators (F_1,69_ = 20.3, *p* = .004). At the univariate level, niche role significantly impacted consumption of small prey (F = 6.07, *p* = .038) and birds (F = 6.49, *p* = .038). Pumas in regions where the species was a subordinate predator consumed fewer small prey than in regions where it was an apex predator. Bird consumption was also higher in systems where the species was an apex predator. Dietary diversity did not change with change in niche role (*p* = .273).

### 3.3 Role of human impacts and time

Our hypothesis regarding impact of human footprint on puma diets was not supported. Human footprint did not significantly affect puma diets (F_1,69_ = 10.36, *p* = .126), including consumption of domestic species and small wild prey species. We did not find support for our prediction regarding increased consumption of domestic species with lower consumption of very large and large wild prey; however, a post hoc analysis suggested that consumption of domestic species decreased with the total consumption of medium‐sized prey (*p* = .027, Spearman's ρ = −.26). A large proportion of studies in our analysis (*n* = 32) did not report any consumption of domestic species. Finally, puma diets did not change with time (F_1,69_ = 10, *p* = .11) and we found no effect of median study year on consumption of very large (F = 0.006, *p* = .928) and large (F = 0.184, *p* = .848) wild species. Detailed results of the multivariate models, including univariate analyses, are available in Appendix S3.

## 4 DISCUSSION

Anthropogenic disturbances have resulted in significant extinctions, local extirpations, and population declines in most taxonomic groups across the world (Ceballos et al., 2015, 2017; IPBES, 2019). Extinction threat levels, however, vary considerably across taxa due to a combination of factors including species’ plasticity and environmental stressors (Young et al., 2016). Diet specialists, for example, are more susceptible to environmental change compared with diet generalists (Clavel et al., 2011; Devictor et al., 2008) with one of the hypotheses for the extinction of the saber‐toothed cats *(Smilodon spp)*, for example, being its dependence on large prey (Meachen‐Samuels & Van Valkenburg, 2010). In this study, we found that at the species level, pumas displayed a high degree of dietary plasticity, consuming an incredible diversity of prey species across their range, ranging from large wild ungulates such as the elk (*Cervus canadensis*), which can weigh over 350 kg, to a multitude of small rodent and mammalian species under a kilogram. At least three studies in our analysis reported pumas switching to other prey species after steep declines in large wild prey numbers (Novaro et al., 2000, Pia, 2013; Sweitzer et al., 1997), including preying on a combination of medium or small native mammals, supplemented by livestock and other exotic species. This remarkable degree of plasticity has important implications for both ecology and conservation.

Dietary plasticity is one mechanism that allows consumers to persist in wide‐ranging environments with variable amounts of disturbances. A recent analysis of carnivores in the neotropics found that pumas have among the greatest range of prey body mass in their diet (Cruz et al., 2022). In comparison, the jaguar, the other large felid in the Americas, is one of the species of felids exhibiting the least amount of variation in prey body mass (Cruz et al., 2022). Given the large differences in dietary flexibility, it is perhaps unsurprising that the jaguar has lost nearly 50% of its original range and is of greater conservation concern than the puma (Nielsen et al., 2015; Zeller, 2007). The ability of pumas to thrive in a wide range of habitats, disperse large distances, fulfill varying roles as an apex or subordinate predator, and survive on a diverse range of prey species is likely to have contributed to its recent resurgence and persistence. Research using dental microwear analysis also suggests that this flexibility may similarly have been critical to the species’ survival through the Late Pleistocene extinction event (DeSantis & Haupt, 2014).

Despite the high level of variability in habitats, trophic positions, and human influence across the puma range, we found clear patterns in puma diet across latitudes, continents, and biomes, in line with results from previous analyses (Iriarte et al., 1990). The consumption of larger prey increased with increasing latitudes, while the consumption of medium prey increased with proximity to the equator. Within continents, the consumption of large species and birds was higher in North America, whereas small species were consumed more in South America. Finally, across biomes pumas had a higher proportion of large and large‐medium prey species in their diet in the desert, grassland, and temperate biomes compared with tropical biomes, where pumas consumed more medium‐sized prey species. In contrast with previous analyses (Iriarte et al., 1990; Murphy & Ruth, 2010), however, dietary diversity did not change across biomes, continents or with the trophic position of pumas. While clear patterns exist, we contend that the observed trends across latitudes, continents, and biomes may likely reflect differences in prey availability. According to optimal foraging theory, pumas should select for prey that maximizes both energy intake and fitness (Stephens & Dunlap, 2007). Selecting for larger prey tends to be more energy efficient, and mammalian predators larger than 21.5 kg in body mass tend to select for prey that are 45% greater than their own body mass, across predator families (Carbone et al., 1999). This behavioral decision is, however, influenced by prey availability and the relative profitability of alternate prey. Predator and prey behavior, morphology, and physiology as well as the landscape in which predation occurs also play a role and may impact the energetic yield (Stephens & Dunlap, 2007). According to Bergmann's rule, animal body size increases with increasing latitudes, which would result in greater availability of larger prey for pumas at higher latitudes (Blackburn et al., 1999). Our results showing greater proportions of larger prey consumed with increasing latitudes and greater proportions of medium prey consumed in tropical biomes are in line with this general rule. Variation in prey size between continents also likely reflects differences in the availability of prey between continents. Almost half of puma diets comprised medium‐sized species in South America as compared to North America, where over half of puma diets comprised large species, with medium‐sized prey accounting for less than a fifth of the species’ diets (Figure 1), although we acknowledge that smaller prey may possibly be overestimated in dietary analyses conducted using frequency‐based methods (Klare et al., 2011) and suggest that the exact proportions reported in our analysis be interpreted with caution. These differences in prey composition may likely be due to a combination of factors including lower large wild prey availability in many parts of the Amazon and the Patagonian steppe and steep declines over time in numbers of many medium‐sized prey species, especially rabbits and hares, in many regions in the puma range in the United States (Ripple et al., 2013). In the Patagonian steppe in southern South America, guanacos represent the only large‐bodied natural prey species for the puma and are often their most important prey item (Walker & Novaro, 2010) and represent a significant proportion of puma diets when abundant (Donadio et al., 2010). With guanacos declining and the species being functionally extinct in many parts of the steppe (Travaini et al., 2015), pumas are reported to rely increasingly on smaller species. Small mammals, including rodents, were thus reported to comprise over 50% of puma diets in several studies from the area (Donadio et al., 2010; Iriarte et al., 1991; Yáñez et al., 1986), with one study reporting that over 80% of puma diets comprised of small mammalian species (Pia, 2013).

Puma diets also varied with their realized niche, with pumas eating more small prey and birds as apex predators. However, puma consumption of larger prey species did not change with their trophic position as apex or subordinate predators. Three possible hypotheses may explain these results. First, the similarities in proportions of larger prey consumed may arise from pumas scavenging on kills made by larger predators, a behavior previously reported from western Canada (Knopff, Knopff, & Boyce, 2010), although other research from the same area reported that pumas showed temporal avoidance of areas used by wolves and did not scavenge from wolf kills (Kortello et al., 2007). Second, while selecting for larger prey tends to be energetically more efficient, this also increases the chances of injury (Mukherjee & Heithaus, 2013). In the absence of larger competing predators, pumas may be opportunistically increasing their intake of smaller prey. Finally, increased consumption of smaller prey and birds may simply reflect increased availability of these species (Iriarte et al., 1990; Murphy & Ruth, 2010). Across their distribution, pumas overlap with several larger predators, including wolves, jaguars, and both black and grizzly bears. As subordinate predators in ecosystems with these larger predators, pumas are likely to partition space, time, or their diet to coexist with more dominant carnivores. The presence of these sympatric predators likely affects puma behavior in multiple ways, including habitat selection and use, which may also consequently affect prey availability or selection (Elbroch & Kusler, 2018). Dietary flexibility may thus be one of several mechanisms by which pumas adapt to changing trophic roles.

In many ecosystems, humans often effectively act as the apex predator, exerting top‐down pressures, including direct and indirect effects, on large predators and lower trophic levels. Although humans have had wide‐ranging impacts on large parts of the puma range across the Americas, our analysis did not show any significant changes in puma diets through time, at least within the broad diet categories used in this analysis, despite large changes in land use in the Americas in the last century. In addition, human impacts on the environment did not appear to directly affect puma diets across categories, countering the hypothesis put forth in a recent study that suggested that puma consumption of large prey was related to levels of human influence (Cruz et al., 2022), although that study was limited to the neotropics. Our result is in line with previous research that reported that pumas continue to take large ungulates even with significant changes in human impacts on the landscape, including urban development (Robins et al., 2019), although research from the Santa Cruz Mountains in California found that pumas increased their predation rates and spent less time at kills in areas with a higher human footprint (Smith et al., 2015, 2017), likely resulting in significant energetic costs. Pumas are thus able to survive in areas with high human impact and activity without significant changes to their diets, albeit by exhibiting behavioral flexibility by changing their predation patterns. We also did not find support for our prediction that puma consumption of domestic species would be correlated with a decrease in consumption of large wild prey. Instead, puma consumption of domestic species was correlated with decreased consumption of medium‐sized species. In combination with our previous findings that pumas appear to consume higher levels of smaller prey as apex predators, we contend that the importance of medium‐sized and smaller prey species to pumas might have been underestimated. While previous studies have reported that domestic species’ consumption by large predators increases with a decrease in wild prey biomass (Khorozyan et al., 2015), we suggest that this relationship may be more nuanced than previously understood. With management of native prey emphasized as a key element of holistic carnivore conflict mitigation strategies (Miller & Schmitz, 2019; Wilkinson et al., 2020), our findings also highlight the importance of maintaining intact native prey assemblages.

Although many studies reported that puma diets were dominated by one primary prey species, pumas demonstrated tremendous variability and differences in diet across regions, habitats, and even individuals, with several interesting results reported by individual studies. Pumas in Alberta, Canada, doubled their intake of non‐ungulate prey species in winter (Knopff, Knopff, Kortello, et al., 2010), in contrast to other findings from Idaho that reported that puma diets in the winter primarily comprised very large and large wild species (Hornocker, 1970). In the Pryor Mountains in Wyoming and Montana in the United States, pumas selected for bighorn sheep (*Ovis canadensis*) (Blake & Gese, 2016), in contrast to findings from studies in neighboring Idaho that reported that bighorn sheep contributions to puma diets were negligible (Hornocker, 1970). In Lihue Calel National Park in Argentina, the plains vizcacha (*Lagostomus maximus*), a large rodent, was the preferred prey for pumas, with pumas increasing consumption of other larger and smaller species only after a steep decline in vizcacha populations during the study (Branch et al., 1996). Pumas in central Argentina preferred large prey over more abundant small rodents, but those in southwestern Argentina preferred European hares (*Lepus europaeus*) even in areas with high guanaco densities (Pia, 2013). Pumas showed a similar trend of higher consumption of an alternative medium‐sized prey, the collared peccary (*Pecari tajacu*), over mule deer (*Odocoileus hemionus*) in another study in Texas (Leopold & Krausman, 1986). These specific examples provide further evidence and insight into puma dietary flexibility, and we contend that multiple factors, including availability, access, and ease of predation affect puma prey selection. Systematic availability of data regarding the background prey species distributions across all study locations, in particular, may significantly aid our understanding of puma prey selection; however, these data are difficult and expensive to obtain in most study systems. Understanding shifts in puma diets toward smaller species may have important implications in terms of persistence of mesopredators and consequent impacts on lower trophic levels in these ecosystems (Crooks & Soulé, 1999).

## 5 CONCLUSIONS & CONSERVATION IMPLICATIONS

Large carnivores play critical roles in terrestrial ecosystems, and research on trophic cascades and issues such as mesopredator release further underlines the importance of conserving functional large carnivore populations (Ripple et al., 2014; Ritchie & Johnson, 2009). Although carnivore populations are often regulated by bottom‐up factors in natural ecosystems (Hayward et al., 2007), humans also both directly and indirectly exert top‐down and bottom‐up pressures, and carnivore diet selection is fundamental to understanding both of these regulating factors. Our results indicating that pumas are dietary generalists give further insight into the success and wide distribution of pumas in comparison with other carnivores (DeSantis & Haupt, 2014; IUCN, 2021) This tremendous dietary flexibility displayed by pumas represents both an opportunity and a challenge.

While pumas may be able to easily adapt to declining availability of their primary prey by prey switching, this likely creates three important conservation and management challenges. One, the ability of pumas to switch to small or alternate prey species, including a large variety of small mammals and rodents, might impact trophic dynamics, including suppressing mesopredator and other small predator communities that are dependent on small prey, and impacts on primary producer communities. Two, this ability to switch to smaller or alternate prey may have implications for puma population dynamics, due to the likely higher energetic costs that will translate into impacts on fitness. Three, the widespread availability of human‐supported subsidies in the form of livestock and other domestic species in large parts of the puma range may result in increased conflict, retaliatory killings, and reduced tolerance for the species. We suggest that future studies explore the linkages between availability and selection of primary and other wild prey and consequent impacts on predation of domestic species to further guide conservation actions and reduce negative interactions between pumas and people.

## AUTHOR CONTRIBUTIONS

**Harshad Karandikar:** Conceptualization (lead); Data curation (equal); Formal analysis (lead); Methodology (equal); Writing – original draft (lead); Writing – review & editing (lead). **Mitchell W Serota:** Data curation (equal); Formal analysis (equal); Methodology (equal); Writing – original draft (supporting); Writing – review & editing (supporting). **Wilson C Sherman:** Data curation (equal); Writing – original draft (supporting); Writing – review & editing (supporting). **Jennifer R Green:** Data curation (equal); Writing – original draft (supporting); Writing – review & editing (supporting). **Guadalupe Verta:** Data curation (equal); Writing – original draft (supporting); Writing – review & editing (supporting). **Claire Kremen:** Formal analysis (supporting); Methodology (supporting); Writing – original draft (supporting); Writing – review & editing (supporting). **Arthur Middleton:** Methodology (supporting); Writing – original draft (supporting); Writing – review & editing (supporting).

## CONFLICT OF INTEREST

The authors declare no conflict of interest.

## Supporting information

AppendixS1

## Data Availability

Manuscripts identified through the literature review process for this analysis are listed in Appendix S1.
