# Supplementary material for: Dietary patterns of a versatile large carnivore, the puma (Puma concolor)
Source: Ecol Evol. 2022 Jun 29;12(6):e9002. doi: 10.1002/ece3.9002 (PMC9240727; doi:10.1002/ece3.9002)
Supplement: Supplementary file 1 — AppendixS1 [file ECE3-12-e9002-s001.doc]

**Dietary patterns of a versatile large carnivore, the puma (*Puma concolor*)

Appendices**

**Appendix 1:** List of studies retained after first set of filters based on the following 5 criteria, outlined in methods - 1) diet data stated FO or PO or either of those metrics could be calculated 2) n>=5, 3) samples were from multiple seasons 4) values for all 7 categories were clearly stated or could be calculated, and 5) puma scats were identified unambiguously. Studies in italics were subsequently dropped based on the sample size criteria outlined in Methods. Multiple sample size values reflect independent data points within the same study.

| **#** | **Study author(s) and year** | **Region** | **Sample Size(s)** |
| --- | --- | --- | --- |
| 1 | Llanos & Travaini 2020 | Argentine Patagonia | 21 |
| *2* | *Tirelli et al. 2018* | *Brazilian Amazon* | *8* |
| 3 | Robins et al. 2019 | Pacific northwest, USA | 568 |
| 4 | Caudill et al. 2019 | Florida, USA | 59, 232 |
| 5 | Ávila-Nájera et al. 2018 | Yucatán Peninsula, Mexico | 26 |
| 6 | Soria-Díaz et al. 2018 | Central Mexico | 150 |
| *7* | *Gelin et al. 2017* | *Central Argentina* | *14* |
| 8 | Zanón Martínez et al. 2016 | Central Argentina | 83 |
| 9 | Iriarte et al. 1991 | Argentine Patagonia | 405 |
| 10 | Rau & Jiménez 2002 | Chilean Patagonia | *3, 12, 11*, 26, *10* |
| 11 | Cassaigne et al. 2016 | Northwestern Mexico | 66, 84 |
| 12 | Allen et al. 2015 | Northern California | 352 |
| 13 | Hernández-SaintMartín et al. 2015 | Northeastern Mexico | 22 |
| 14 | Fernandez & Baldi 2014 | Argentine Patagonia | 71 |
| *15* | *Chinchilla 1997* | *Costa Rica* | *11* |
| 16 | Elbroch et al. 2013 | Yellowstone ecosystem, USA | 650 |
| 17 | Nuñez et al. 2000 | Central Mexico | 65 |
| 18 | Cunningham et al. 1999 | Southeast Arizona, USA | 370 |
| *19* | *Reuda et al. 2013* | *Central Mexico* | *19* |
| 20 | Zúñiga & Muñoz-Pedredos 2013 | Southern Chile | 55 |
| *21* | *Aranda & Sánchez-Cordero 1996* | *Southeastern Mexico* | *15* |
| 22 | Novack et al. 2005 | Northern Guatemala | 82, 63 |
| 23 | Leopold & Krausman 1986 | Southeastern mexico | 161, 272 |
| 24 | Bacon 2010 | Alberta and Saskatchewan, Canada | 211, 266 |
| 25 | Anderson & Lindzey 2003 | Wyoming, USA | 74 |
| 26 | Wilckens et al. 2016 | North Dakota, USA | 292 |
| 27 | Toweill & Maser 1985 | Oregon, USA | 61, 54 |
| 28 | Zanón Martínez et al. 2012 | Argentine Patagonia | 204, 63, 15 |
| 29 | Franklin et al. 1998 | Southern Chile | 405 |
| 30 | Monroy-Vilchis et al. 2009 | Central Mexico | 104 |
| 31 | Gómez-Ortiz & Monroy-Vilchis 2013 | Central Mexico | 209 |
| 32 | Cashman et al. 1992 | Southwestern Arizona, USA | 159 |
| 33 | Spalding & Lesowski 1971 | British Columbia, Canada | 62, 37 |
| *34* | *Ludwig et al. 2007* | *Southern Brazil* | *10* |
| 35 | Maehr et al. 1990 | Southwest Florida, USA | 270 |
| 36 | Toweill & Meslow 1977 | Oregon, USA | 25 |
| 37 | de la Torre & de la Riva 2009 | Central Mexico | 38 |
| 38 | Taber et al. 1997 | Centralwest and Northwest Paraguay | 95 |
| 39 | Kerston et al. 2011 | Pacific Northwest, USA | 304 |
| 40 | Pacheco Jaimes et al. 2018 | Eastern Andes, Colombia | 45 |
| 41 | Polisar et al. 2003 | North-central Venezuela | 42, *5* |
| *42* | *Lowrey et al. 2016* | *Colorado, USA* | *7* |
| 43 | Santos et al. 2014 | South-eastern Brazil | 27 |
| *44* | *Thompson et al. 2009* | *North & South Dakota, USA* | *14* |
| 45 | Rosas-Rosas et al. 2003 | Northwestern Mexico | 60 |
| *46* | *Emmons 1987* | *Southeastern Peru* | *7* |
| 47 | Hass 2009 | South-eastern Arizona, USA | 64 |
| 48 | Hernandez-Guzman et al. 2011 | South Colombian Andes | 60 |
| 49 | Robinette et al. 1959 | Utah and Nevada, USA | 275, 277 |
| 50 | Elbroch et al. 2013 | Chilean Patagonia | 462 |
| 51 | Harveson et al. 2000 | Southern Texas, USA | 25, 75 |
| 52 | Yanez et al. 1986 | Chilean Patagonia | 439, 243 |
| 53 | Knopff et al. 2010 | Alberta, Canada | 1428 |
| 54 | Blake & Gese 2016 | Montana, USA | 200 |
| 55 | Nichols 2017 | Central California, USA | 250 |
| 56 | Branch et al. 1996 | Northern Argentina | 268, 67 |
| 57 | Pittman et al. 2000 | Western Texas, USA | 19, 135 |
| 58 | Ackerman et al. 1984 | South-central Utah, USA | 239, 112 |
| 59 | Schwartz & Mitchell 1945 | Pacific northwest, USA | 28 |
| 60 | De Azevedo 2008 | Southern Brazil | 54 |
| *61* | *Martins et al. 2008* | *Brazil* | *12* |
| 62 | Foster et al. 2010 | Belize | 135 |
| 63 | Zapata et al. 2007 | Argentine Patagonia | 39 |
| 64 | Novaro et al. 2000 | Argentine Patagonia | 70 |
| 65 | Gomez-Ortiz et al. 2011 | Southwestern Mexico | 183 |
| 66 | Crawley and Quigley 2002 | Pantanal, Brazil | 28 |
| *67* | *Brito 2000* | *Southern Brazil* | *12* |
| 68 | Vidolin 2004 | South-eastern Brazil | 43 |
| 69 | Moreno et al. 2006 | Central Panama | 88 |
| 70 | Villepique et al. 2011 | Central California | 252 |
| 71 | Pia 2013 | Argentine Patagonia | 234 |
| 72 | Donadio et al. 2010 | Multiple sites in Argentina | 366, *10, 8, 7,* 31, *18,* 20 |
| 73 | Rosas-Rosas et al. 2008 | Northern mexico | 88 |

**Appendix 2:** List of reported puma prey species, listed by category (D: Domestic species, VL: Very large, L: Large, M: Medium, SM: Small mammals, SR: Small rodents (listed separately from Small mammals but combined in the analysis, B: Birds and O: Other species)

| **#** | **Common name** | **Scientific name** | **Category** |
| --- | --- | --- | --- |
| 1 | Chicken | *Gallus gallus domesticus* | D |
| 2 | Cow, cattle | *Bos taurus* | D |
| 3 | Domestic cat | *Felis catus* | D |
| 4 | Dog | *Canis lupus familiaris* | D |
| 5 | Donkey, ass | *Equus asinus* | D |
| 6 | Goat | *Capra aegagrus hircus* | D |
| 7 | Llama | *Lama glama* | D |
| 8 | Sheep | *Ovis aries* | D |
| 9 | Elk, Red deer | *Cervus canadensis, Cervus elaphus* | VL |
| 10 | Horse | *Equus ferus caballus* | VL |
| 11 | Moose | *Alces alces* | VL |
| 12 | Bighorn sheep | *Ovis canadensis* | L |
| 13 | Black bear | *Ursus americanus* | L |
| 14 | Blackbuck | *Antilope cervicapra* | L |
| 15 | Capybara | *Hydrochoerus hydrochaeris* | L |
| 16 | Chacoan peccari | *Catagonus wagneri* | L |
| 17 | Giant Anteater | *Myrmecophaga tridactyla* | L |
| 18 | Gray wolf | *Canis lupus* | L |
| 19 | Guanaco | *Lama guanicoe* | L |
| 20 | Huemul, South andean deer | *Hippocamelus bisulcus* | L |
| 21 | Mountain goat | *Oreamnos americanus* | L |
| 22 | Mule deer | *Odocoileus hemionus, Odocoileus hemionus crooki* | L |
| 23 | Pronghorn | *Antilocapra americana* | L |
| 24 | Puma, Cougar, Mountain lion | *Puma concolor, felis concolor* | L |
| 25 | White-lipped peccary | *Tayassu pecari* | L |
| 26 | White-tailed deer | *Odocoileus virginianus* | L |
| 27 | Wild boar, Wild Hog | *Sus scrofa* | L |
| 28 | American badger | *Taxidea taxus* | M |
| 29 | American beaver | *Castor canadensis* | M |
| 30 | American Hog Nosed Skunk | *Conepatus leuconotus* | M |
| 31 | Antelope jackrabbit | *Lepus alleni* | M |
| 32 | Azara's Agouti | *Dasyprocta azarae* | M |
| 33 | Big hairy armadillo, Patagonian haired armadillos | *Chaetophractus villosus* | M |
| 34 | Black capuchin | *Sapajus nigritus, Cebus nigritus* | M |
| 35 | Black-handed spider monkey | *Ateles geoffroyi* | M |
| 36 | Black howler monkey | *Alouatta caraya* | M |
| 37 | Bobcat | *Lynx rufus, Felis rufus* | M |
| 38 | Brown agouti | *Dasyprocta variegata* | M |
| 39 | Brown howler | *Alouatta guariba, Alouatta fusca* | M |
| 40 | Brown throated three toed sloth | *Bradypus variegatus* | M |
| 41 | Canada lynx | *Lynx canadensis* | M |
| 42 | Central American red brocket | *Mazama temama* | M |
| 43 | Collared peccary, Javelina | *Pecari tajacu, Tayassu tacaju* | M |
| 44 | Colombian white-faced capuchin | *Cebus capucinus* | M |
| 45 | Common agouti | *Dasyprocta agouti* | M |
| 46 | Coyote | *Canis latrans* | M |
| 47 | Coypu | Myocastor coypus | M |
| 48 | Crab-eating fox | *Cerdocyon thous* | M |
| 49 | Crab-eating racoon | *Procyon cancrivorus* | M |
| 50 | Culpeo | *Lycalopex culpaeus, Dusicyon culpaeus, Pseudalopex culpaeus* | M |
| 51 | Dwarf Red Brocket | Mazama rufina | M |
| 52 | European hare | *Lepus europaeus* | M |
| 53 | Fisher | *Pekania pennanti* | M |
| 54 | Geoffroy's cat | *Leopardus geoffroyi, Oncifelis geoffroyi* | M |
| 55 | Gray brocket | *Mazama gouazoubira* | M |
| 56 | Gray fox | *Urocyon cinereoargenteus* | M |
| 57 | Greater naked-tailed armadillo | *Cabassous tataouay* | M |
| 58 | Guatemalan black howler monkey | *Alouatta pigra* | M |
| 59 | Hoffman's two-toed sloth | *Choloepus hoffmanni* | M |
| 60 | Humbolt's hog nosed skunk | *Conepatus humboldtii* | M |
| 61 | Jaguarundi | *Puma yagouaroundi* | M |
| 62 | Lowland paca, Agouti | *Cuniculus paca, Agouti paca* | M |
| 63 | Mantled howler monkey | *Alouatta palliata* | M |
| 64 | Margay | *Leopardus wiedii* | M |
| 65 | Mexican Cottontail | Sylvilagus cunicularius | M |
| 66 | Mexican hairy-dwarfed porcupine | *Sphiggurus mexicanus* | M |
| 67 | Molina's hog-nosed skunk | *Conepatus chinga* | M |
| 68 | Mountain Paca | *Cuniculus taczanowskii* | M |
| 69 | Nine-banded armadillo | *Dasypus novemcinctus* | M |
| 70 | North American porcupine | *Erethizon dorsatum* | M |
| 71 | North American river otter | *Lontra canadensis* | M |
| 72 | Northern muriqui | *Brachyteles hypoxanthus* | M |
| 73 | Northern pudu | Pudu mephistophiles | M |
| 74 | Northern tamandua | *Tamandua mexicana* | M |
| 75 | Ocelot | *Leopardus pardalis* | M |
| 76 | Pampas cat, Colocolo | *Leopardus colocola, Oncifelis colocolo* | M |
| 77 | Pampas fox | *Lycalopex gymnocercus* | M |
| 78 | Patagonian mara | *Dolichotis patagonum* | M |
| 79 | Plains vizcacha | *Lagostomus maximus* | M |
| 80 | Raccoon, Common raccoon | *Procyon lotor* | M |
| 81 | Red brocket | *Mazama americana* | M |
| 82 | Red-rumped agouti | *Dasyprocta leporina* | M |
| 83 | Red fox | *Vulpes vulpes* | M |
| 84 | Southern american coati | *Nasua nasua* | M |
| 85 | Southern Pudu | *Pudu pudu, Pudu Puda* | M |
| 86 | Southern Tamandua | *Tamandua tetradactyla* | M |
| 87 | Striped skunk | *Mephitis mephitis* | M |
| 88 | Tayra | *Eira barbara* | M |
| 89 | Tufted capuchin | *Cebus apella* | M |
| 90 | Yellow armadillo | *Euphractus sexcinctus* | M |
| 91 | Yellow-bellied marmot | *Marmota flaviventris* | M |
| 92 | Western mountain coati | Nasuella olivacea | M |
| 93 | White-nosed coati | *Nasua narica* | M |
| 94 | White-tailed jackrabbit | *Lepus townsendii* | M |
| 95 | American marten, American pine marten | *Martes americana* | SM |
| 96 | American mink | *Neovison vison, Mustela vision* | SM |
| 97 | Big-eared opossum | *Didelphis aurita* | SM |
| 98 | Black-tailed jackrabbit | *Lepus californicus* | SM |
| 99 | Brazilian cottontail, Tapeti | *Sylvilagus brasiliensis* | SM |
| 100 | Brush rabbit | *Sylvilagus bachmani* | SM |
| 101 | Cape hare | *Lepus capensis* | SM |
| 102 | Chacoan mara | *Dolichotis salinicola* | SM |
| 103 | Common opossum | *Didelphis marsupialis* | SM |
| 104 | Desert cottontail | *Sylvilagus audubonii* | SM |
| 105 | Eastern cottontail | *Sylvilagus floridanus* | SM |
| 106 | Eastern Spotted Skunk | *Spilogale putorius* | SM |
| 107 | European Rabbit | *Oryctolagus cuniculus* | SM |
| 108 | Gophers | *Geomyidae sp.* | SM |
| 109 | Grey four-eyed opossum | *Philander opossum* | SM |
| 110 | Grayish mouse opossum | *Tlacuatzin canescens, Marmosa canescens* | SM |
| 111 | Hooded Skunk | *Mephitis macroura* | SM |
| 112 | Kinkajou | *Potos flavus* | SM |
| 113 | Lesser grison | *Galictis cuja* | SM |
| 114 | Long-nosed short-tailed opossum | *Monodelphis scalops* | SM |
| 115 | Long-tailed weasel | *Mustela frenata* | SM |
| 116 | Monito del monte | *Dromiciops gliroides* | SM |
| 117 | Nuttall's cottontail | *Sylvilagus nuttallii* | SM |
| 118 | Paraguaian hairy-dwarf porcupine | *Coendou spinosus, Sphiggurus villosus* | SM |
| 119 | Patagonian Opossum | Lestodelphys halli | SM |
| 120 | Pichi, Dwarf armadillo, Pygmy armadillo | *Zaedyus pichiy* | SM |
| 121 | Ring-tailed cat | *Bassariscus astutus* | SM |
| 122 | Snowshoe hare | *Lepus americanus* | SM |
| 123 | South American vesper bats | *Histiotus sp.* | SM |
| 124 | Southeastern four-eyed opossum | *Philander frenatus* | SM |
| 125 | Southern three banded armadillo | *Tolypeutes matacus* | SM |
| 126 | Southern viscacha | *Lagidium viscacia* | SM |
| 127 | Trowbridge shrew | *Sorex trowbridgii* | SM |
| 128 | Virginia opossum | *Didelphis virginiana* | SM |
| 129 | Western spotted skunk | *Spilogale gracilis* | SM |
| 130 | White-bellied fat-tailed mouse opossum | *Thylamys pallidior* | SM |
| 131 | White eared opossum | *Didelphis albiventris* | SM |
| 132 | (Unknown) | *Abrotrix xanthorinus* | SR |
| 133 | (Unknown) | *Cricetidae spp.* | SR |
| 134 | Amazonian hocicudo | *Oxymycterus amazonicus* | SR |
| 135 | American red squirrel | *Tamiasciurus hudsonicus* | SR |
| 136 | Bunny rat | *Reithrodon auritus* | SR |
| 137 | Botta's pocket gopher | *Thomomys bottae* | SR |
| 138 | Central american agouti | *Dasyprocta punctata* | SR |
| 139 | Chipmunks | *Tamias, Sciuridae* | SR |
| 140 | California ground squirrel | *Otospermophilus beecheyi* | SR |
| 141 | Common yellow-toothed cavy | *Galea musteloides* | SR |
| 142 | Cursor grass mouse | *Akodon cursor* | SR |
| 143 | Darwin's leaf eared mouse | *Phyllotis darwini* | SR |
| 144 | Desert woodrat | *Neotoma lepida* | SR |
| 145 | Douglas squirrel | *Tamiasciurus douglasii* | SR |
| 146 | Flying squirrel, Northern flying squirrel | *Glaucomys sabrinus* | SR |
| 147 | Golden-mantled ground squirrel | *Callospermophilus lateralis, Spermophilus lateralis* | SR |
| 148 | Gray leaf-eared mouse | *Graomys griseoflavus* | SR |
| 149 | Ground squirrel | *Citellus sp.* | SR |
| 150 | Guinea pigs | *Cavia porcellus* | SR |
| 151 | Hairy-tailed bolo mouse | *Necromys lasiurus* | SR |
| 152 | Highland gerbil mouse | *Eligmodontia typus* | SR |
| 153 | Hispid cotton rat | *Sigmodon hispidus* | SR |
| 154 | Hoary marmot | *Marmota caligata* | SR |
| 155 | House mouse | *Mus musculus* | SR |
| 156 | House rat | Rattus rattus | SR |
| 157 | Intelligent grass mouse, Patagonian akodont | *Akodon iniscatus* | SR |
| 158 | jumping mouse | *Zapus sp.* | SR |
| 159 | Little pocked mouse | *Perognathus longimembris* | SR |
| 160 | Long Haired Grass Mouse, Long Haired Akodont | *Abrothrix longipilis* | SR |
| 161 | Long-tailed colilargo, Long-tailed pygmy rice rat | *Oligoryzomys longicaudatus, Oryzomys longicaudatus* | SR |
| 162 | Marsh Rice Rat | Oryzomys palustris | SR |
| 163 | Meadow mouse | *Microtus pennsylvanicus* | SR |
| 164 | Megellanic tuco-tuco | *Ctenomys magellanicus* | SR |
| 165 | Mendoza tuco-tuco | *Ctenomys mendocinus* | SR |
| 166 | Mexican grey squirrel | *Sciurus aureogaster* | SR |
| 167 | Mexican spiny pocket mouse | *Liomys irroratus, Heteromys irroratus* | SR |
| 168 | Molinas Grass Mouse | *Akodon molinae* | SR |
| 169 | Morgan's gerbil mouse, western Patagonian laucha | *Eligmodontia morgani* | SR |
| 170 | Mountain beaver | *Aplodontia rufa* | SR |
| 171 | Muskrat | *Ondatra zibethicus* | SR |
| 172 | Olive Grass Mouse | *Abrothrix olivaceus* | SR |
| 173 | Pine squirrel | *Sciurus douglas* | SR |
| 174 | Red tailed squirrel | *Sciurus granatensis* | SR |
| 175 | Rock squirrel | *Otospermophilus variegatus, Spermophilus variegatus* | SR |
| 176 | (Unknown) | *Reithrodon physodes* | SR |
| 177 | South American spiny rats | *Proechimys semispinosus* | SR |
| 178 | Southern big-eared mouse | *Loxodontomys micropus, Auliscomys micropus* | SR |
| 179 | Southern mountain cavy | *Microcavia australis* | SR |
| 180 | Spiny rats | *Echimyidae sp.* | SR |
| 181 | Vesper mouse | *Calomys sp.* | SR |
| 182 | Western gray squirrel | *Sciurus griseus* | SR |
| 183 | White-footed mouse | *Peromyscus leucopus* | SR |
| 184 | White-tailed antelope squirrel | *Ammospermophilus leucurus* | SR |
| 185 | Wood rat | *Neotoma sp.* | SR |
| 186 | Yellow-rumped leaf-eared mouse | *Phyllotis xanthopygus* | SR |
| 187 | American robin | *Turdus migratorius* | B |
| 188 | Anatidae sp. | *Anatidae sp.* | B |
| 189 | Black Necked Swan | *Cygnus melancoryphus* | B |
| 190 | California quail | *Callipepla californica* | B |
| 191 | California towhee | *Melozone crissalis* | B |
| 192 | Canada goose | *Branta canadensis* | B |
| 193 | Common raven | *Corvus corax* | B |
| 194 | Darwin's Rhea, Lesser Rhea | *Rhea pennata* | B |
| 195 | Eared Dove | *Zenaida auriculata* | B |
| 196 | Great curassow | *Crax rubra* | B |
| 197 | Greater Rhea | *Rhea americana* | B |
| 198 | Great-horned owl | *Bubo virginianus* | B |
| 199 | Grouse | *Tetraoninae sp.* | B |
| 200 | Mallard | *Anas platyrhynchos* | B |
| 201 | Mountain quail | *Oreortyx pictus* | B |
| 202 | Mourning dove | *Zenaida macroura* | B |
| 203 | New world vultures, condors | *Catharidae* | B |
| 204 | Ocellated turkey | *Agriocharis ocellatata, Meleagris ocellata* | B |
| 205 | Patagonian Tinamou | *Tinamotis ingoufi* | B |
| 206 | Pheasants | *Galliformes sp.* | B |
| 207 | Plains chachalaca | *Ortalis vetula* | B |
| 208 | Red-naped sapsucker | *Sphyrapicus nuchalis* | B |
| 209 | Ruffed grouse | *Bonasa umbellus* | B |
| 210 | Snowy egret | *Egretta thula* | B |
| 211 | Solitary tinamou | *Tinamus solitarius* | B |
| 212 | Tanagers | *Thraupinae sp.* | B |
| 213 | Tawny-breasted Tinamou | *Nothocercus julius* | B |
| 214 | Upland goose | *Chloephaga picta* | B |
| 215 | West Mexican Chachalaca | Ortalis poliocephala | B |
| 216 | Wild turkey | *Meleagris gallopavo, Mellagris gallopavo* | B |
| 217 | American alligator | *Alligator mississippiensis* | O |
| 218 | Argentine black and white tegu | *Salvator merianae, Tupinambis merianae* | O |
| 219 | Broad-snouted Caiman | *Caiman latirostris* | O |
| 220 | Chaco tortoise | *Geochelone chilensis* | O |
| 221 | Chuckwalla | *Sauromalus obesus* | O |
| 222 | Colubrid snakes | *Colubridae sp.* | O |
| 223 | Desert Tortoise | *Gopherus agassizii* | O |
| 224 | Gila Monster | Heloderma suspectum | O |
| 225 | Golden Tegu | *Tupinambis teguixin* | O |
| 226 | Iguana | *Iguana iguana* | O |
| 227 | Mexican mud turtle | *Kinosternon integrum* | O |
| 228 | Mexican spiny-tailed iguana | *Ctenosaura pectinata* | O |
| 229 | Southern mud turtle | *Kinosternon sonoriense* | O |
| 230 | Spectacled caiman | *Caiman crocodilus* | O |
| 231 | Vipers | *Viperidae sp.* | O |
| 232 | Whiptails | *Teiidae sp.* | O |

**Appendix 3:** Results of multivariate models used for analyzing effects of latitudes, biomes, continents, niche roles and time on puma diets. Significant results are highlighted in bold.

Effect of latitude on puma diet

Multivariate results:

| Res.Df | Df.diff | Dev | Pr(>Dev) |
| --- | --- | --- | --- |
| 69 | 1 | 42.51 | **0.001** |

Univariate results:

| Predictor variable | Response variables | Dev | Pr(>Dev) |
| --- | --- | --- | --- |
| Latitude | Domestic species | 0.004 | 0.961 |
|  | Very large | 15.764 | **0.001** |
|  | Large | 8.867 | **0.007** |
|  | Medium | 15.258 | **0.001** |
|  | Small, including rodents | 0.059 | 0.959 |
|  | Birds | 0.457 | 0.833 |
|  | Others | 2.099 | 0.394 |

Effect of biomes on puma diet

Multivariate results:

| Res.Df | Df.diff | Dev | Pr(>Dev) |
| --- | --- | --- | --- |
| 66 | 4 | 93.82 | **0.001** |

Univariate results:

| Predictor variable | Response variables | Dev | Pr(>Dev) |
| --- | --- | --- | --- |
| Biome | Domestic species | 8.139 | 0.202 |
|  | Very large | 17.254 | **0.008** |
|  | Large | 21.886 | **0.002** |
|  | Medium | 27.08 | **0.001** |
|  | Small, including rodents | 2.198 | 0.609 |
|  | Birds | 13.154 | **0.045** |
|  | Others | 4.103 | 0.562 |

Effect of continent on puma diet

Multivariate results:

| Res.Df | Df.diff | Dev | Pr(>Dev) |
| --- | --- | --- | --- |
| 69 | 1 | 39.91 | **0.001** |

Univariate results:

| Predictor variable | Response variables | Dev | Pr(>Dev) |
| --- | --- | --- | --- |
| Continent | Domestic species | 0.449 | 0.701 |
|  | Very large | 2.709 | 0.243 |
|  | Large | 12.865 | **0.007** |
|  | Medium | 2.432 | 0.243 |
|  | Small, including rodents | 8.694 | **0.010** |
|  | Birds | 12.524 | **0.007** |
|  | Others | 0.233 | 0.701 |

Effect of niche roles on puma diet

Multivariate results:

| Res.Df | Df.diff | Dev | Pr(>Dev) |
| --- | --- | --- | --- |
| 69 | 1 | 20.3 | **0.004** |

Univariate results:

| Predictor variable | Response variables | Dev | Pr(>Dev) |
| --- | --- | --- | --- |
| Niche role | Domestic species | 0.017 | 0.916 |
|  | Very large | 5.133 | **0.040** |
|  | Large | 1.309 | 0.546 |
|  | Medium | 0.102 | 0.916 |
|  | Small, including rodents | 6.072 | **0.033** |
|  | Birds | 6.485 | **0.029** |
|  | Others | 1.185 | 0.553 |

Effect of footprint on puma diet

Multivariate results:

| Res.Df | Df.diff | Dev | Pr(>Dev) |
| --- | --- | --- | --- |
| 69 | 1 | 10.36 | 0.126 |

Univariate results:

| Predictor variable | Response variables | Dev | Pr(>Dev) |
| --- | --- | --- | --- |
| Footprint index value | Domestic species | 0.888 | 0.770 |
|  | Very large | 1.31 | 0.731 |
|  | Large | 3.853 | 0.212 |
|  | Medium | 2.793 | 0.397 |
|  | Small, including rodents | 0.584 | 0.805 |
|  | Birds | 0.462 | 0.805 |
|  | Others | 0.47 | 0.805 |

Effect of time on puma diet

Multivariate results:

| Res.Df | Df.diff | Dev | Pr(>Dev) |
| --- | --- | --- | --- |
| 69 | 1 | 9.996 | 0.109 |

Univariate results:

| Predictor variable | Response variables | Dev | Pr(>Dev) |
| --- | --- | --- | --- |
| Median year | Domestic species | 1.53 | 0.607 |
|  | Very large | 0.006 | 0.914 |
|  | Large | 0.184 | 0.847 |
|  | Medium | 4.159 | 0.168 |
|  | Small, including rodents | 0.844 | 0.618 |
|  | Birds | 1.642 | 0.607 |
|  | Others | 1.630 | 0.607 |
